# 측정도구의 심리계량적 속성 4: 신뢰도와 반응도

**DOI:** 10.4069/kjwhn.2021.11.01

**Published:** 2021-11-25

**Authors:** Eun-Hyun Lee

**Affiliations:** Graduate School of Public Health, Ajou University, Suwon, Korea; 아주대학교 보건대학원

**Keywords:** Reliability, Psychometrics, Responsiveness, 신뢰도, 심리계량, 반응도

## Abstract

For test-retest reliability, it is important to consider whether the attributes of the construct being measured is temporally stable or not. For responsiveness, it needs a longitudinal study design including a treatment/intervention known to induce a change on the construct to be measured.

## 서론

측정도구의 심리계량적 속성에 대한 시리즈의 마지막 편으로 신뢰도(reliability)와 반응성(responsiveness)에 대해 알아보고자 한다.

## 신뢰도

신뢰도는 측정이 얼마나 재현될 수 있는가를 의미한다. 즉, 측정점수들이 얼마나 관련이 있는지 그 정도뿐 아니라, 얼마나 일치하는지를 반영하는 것이다[1]. 수학적으로는 참 점수(true score) 변량에 대한 관찰 점수(observed score: 참 점수 + 측정오차) 변량의 비를 의미한다. 측정오차가 적을수록 측정에 대한 신뢰성이 더 좋다고 할 수 있다[2].

신뢰도의 종류는 평가자 간 신뢰도(interrater reliability), 검사-재검사 또는 반복측정 신뢰도(test-retest reliability) 및 평가자 내 신뢰도(intrarater reliability)로 나눌 수 있다. 평가자 간 신뢰도는 같은 연구대상자를 대상으로 2명 이상의 평가자가 측정한 점수 간의 변이를 의미한다. 검사-재검사 신뢰도는 측정도구의 안전성(stability)을 보기 위한 것으로, 동일한 도구를 가지고 같은 조건 하에 있는 같은 대상자의 어떤 특성을 2번 이상 반복해서 측정하였을 때 얻어지는 결과가 일관되는지를 평가하는 것이다. 평가자 내 신뢰도는 1명의 평가자가 여러 번 반복해서 측정했을 때의 점수의 변이를 말한다. 신뢰도 검정에서 가장 많이 사용되는 연속형 측정점수의 검사-재검사 신뢰도에 대해 조금 더 자세히 살펴보면 다음과 같다.

설문지로 측정된 점수가 연속형인 경우, 검사-재검사 신뢰도 검정을 위한 지표는 급내상관계수(intraclass correlation coefficient, ICC)를 사용한다. 흔히 사용하는 SPSS 프로그램을 사례로 들면, 검사-재검사 신뢰도 검정을 위한 모델로는 “2-way mixed-effects”를 선택하고, 유형으로는 “consistency”를 지정하며, 신뢰구간은 95%로 지정한다. 분석 결과 단일측도(single measures) 및 평균측도(average measures)에 대한 ICC 수치와 95% 신뢰구간이 제시된다. 만약 검사-재검사 신뢰도 검정을 위해 1차와 2차 측정의 평균값이 사용되었다면, 평균측도의 ICC와 이에 대한 신뢰구간을 보면 된다. 이론적으로 ICC의 범위는 0에서 1.0이며, 일반적으로 .70 이상이면 신뢰도를 만족하였다고 할 수 있다[2].

검사-재검사 신뢰도 검정에서 고려해야 할 사항으로는 측정과 재측정 사이의 시간 간격(time interval)이 있다. 시간 간격을 설정할 때 특별히 정해진 바는 없지만, 응답한 기억을 소환하지 않을 정도로 충분한 간격이어야 함과 동시에 대상자의 상황 및 조건이 변하지 않을 정도의 시간 간격이어야 한다. 측정되는 구성개념의 특성 또한 고려해야 할 사항이다. 만약에 구성개념이 시시각각으로 변화하는 특성을 가지고 있다면, 검사-재검사 검정을 하지 말아야 한다. 예를 들어 Lee 등[3]은 우울, 불안 및 스트레스를 측정하는 정서적 측정도구의 심리계량적 속성 연구에서 검사-재검사 신뢰도를 실시하지 않았다고 밝혔는데, 그 이유로는 속성이 검사-재검사 신뢰도를 실시하기에 안정적(stable) 속성을 가진 구성개념이 아니기 때문이라고 하였다.

## 반응성

반응성이란 측정된 구성개념이 시간의 흐름에 따라 변화는 것을 감지해내는 능력을 의미한다[4]. 반응성 검정에서 구성개념 점수의 변화를 보기 위해서는 최소한 두 번 이상 측정하는 종적연구설계가 필수적이다. 종적연구설계에는 측정하고자 하는 구성개념 점수의 변화를 향상 또는 악화시킨다고 입증된 처치나 중재 제공이 포함되어야 한다. 그리고 실제로 검증하고자 하는 도구로 측정한 점수가 연구자가 기대한 것처럼 향상 또는 악화되었는지를 판단하는 것이다. Lee 등[5]은 한 간호학 학술지에 게재된 측정도구의 심리계량적 속성 연구논문을 고찰한 결과 반응성을 검정한 논문이 없었음을 확인하고, 앞으로 측정도구 연구에서 반응성도 함께 검정할 수 있는 종적연구를 계획할 것을 권유하였다.

반응성을 검정하기 위해 사용되는 분석방법으로는 효과 크기(effect size; mean change score/standard deviation [SD] baseline), 표준평균 반응도(standardized response mean [SRM]; mean change score/SD change score), 반응도 계수(Norman’s responsiveness coefficient; σ^2^ change/[σ^2^ change+σ^2^ error]), 상대적 유효성 통계량(relative efficacy statistic; [t-statistic_1_/t-statistic_2_]^2^)이 있다[6-9]. 하지만 반응성 검정에 자주 등장하는 대응 t-검정은 변화의 유의성을 측정하는 것이지 변화의 타당성을 검증하는 것이 아니므로 측정도구의 반응성 검증방법으로 적합하지 않다[10].

반응성 검정 사례를 살펴보면, 천식 특이형 삶의 질 측정도구 개발에서 Lee 등[11]은 천식 진단을 받고 아직 치료를 받지 않은 신환 환자를 대상으로 개발한 삶의 질 측정도구를 사용해서 점수를 측정하고, 치료방법으로 약물을 복용 후 1개월 후에 다시 삶의 질을 측정하였다. 그리고 SRM 분석을 통해서 개발한 측정도구의 반응성을 검정하였다.

## 요약

검사-재검사 신뢰도 검정을 할 때는 측정하고자 하는 구성개념에 대한 속성을 주의 깊게 파악해서 검사-재검사 검정이 필요한지 확인하는 것이 중요하다. 앞으로 측정도구 개발연구에서 반응성 검정을 위해 종적연구설계를 계획할 것을 권유한다.
